# Large Intra-Articular Anterior Cruciate Ligament Ganglion Cyst, Presenting with Inability to Flex the Knee

**DOI:** 10.1155/2010/705919

**Published:** 2011-01-10

**Authors:** Jake Sloane, Vivek Gulati, Sreeram Penna, Philip Pastides, Davinder Paul Singh Baghla

**Affiliations:** ^1^Department of Orthopaedics, Ealing Hospital, Southall, London UB13HW, UK; ^2^Department of Orthopaedics, Northwick Park Hospital, Harrow, London HA13UJ, UK

## Abstract

A 41-year-old female presented with a 3-month history of gradually worsening anterior knee pain, swelling and inability to flex the knee. Magnetic resonance imaging (MRI) revealed a large intra-articular cystic swelling anterior to the anterior cruciate ligament (ACL), extending into the Hoffa's infrapatellar fat pad. Following manipulation under anaesthesia and arthroscopic debridement of the cyst, the patient's symptoms were relieved with restoration of normal knee motion. ACL ganglion cysts are uncommon intra-articular pathological entities, which are usually asymptomatic and diagnosed incidentally by MRI. This is the first reported case of an ACL cyst being so large as to cause a mechanical block to knee flexion.

## 1. Introduction

A ganglion is a cystic tumour-like lesion filled with gelatinous fluid containing hyaluronic acid and other mucopolysaccharides, surrounded by a dense network of collagen fibres and fibrocytes [1]. Ganglions usually arise from tendon sheaths, joint capsules or muscles and can be solitary or multilobulated [2].

Although the dorsum of the hand is the most common location [3], ganglion cysts around the knee are not infrequent. The majority of cystic lesions around knee are extra-articular meniscal cysts [1]. These meniscal cysts may arise from meniscal tear and are different from commonly seen ganglion cysts. Intra-articular ganglion cysts arising from the cruciate ligaments are rarer still. Most patients with intra-articular ganglion cysts are asymptomatic with diagnoses made incidentally on MRI or routine arthroscopy [4]. Clinical symptoms associated with complicated ganglion cysts include pain, stiffness, and mechanical locking [5–7]. It is reported that ganglia arising anterior to the anterior cruciate ligament (ACL) tend to limit knee extension, whilst those occurring posterior to the posterior cruciate ligament (PCL) limit knee flexion [4]. We uniquely report a symptomatic extensive ACL ganglion cyst extending into Hoffa's fat pad, presenting with pain and inability to flex the knee-“reverse locking.” The differential diagnoses of patients presenting with such pain and stiffness include all causes for a subacute internal derangement within the knee whether it is post traumatic, inflammatory, or degenerative.

## 2. Case Presentation

A 41-year-old Caucasian female presented with a two-month progressive history of left-sided anterior knee pain, associated with an inability to fully flex the knee. She attributed her symptoms to an initial minor twisting injury whilst standing up from a kneeling position. Interestingly she admitted to spend considerable time kneeling down on the floor both at work as a teacher as well as recreationally. Paracetamol and Ibuprofen provided only temporary pain relief, whilst physiotherapy had exacerbated her pain and stiffness. There was no significant past medical history nor any previous knee symptoms. She eventually presented to the senior author (DB) 8 weeks following the onset of her symptoms.

Clinical examination revealed her left knee to be held in an extension posture. No joint effusion was noted, but a firm tender swelling was palpable along the anterolateral knee joint line. Mild tenderness was also noted at the lateral patellofemoral joint. Range of both active as well as passive flexion was limited to 40 degrees, whereas she could extend normally to 5 degree hyperextension. No ligamentous instability was noted, and McMurray's test for meniscal pathology was too painful to perform. We describe this clinical presentation as “reverse locking,” implying a mechanical block to knee flexion.

**Figure 1:**
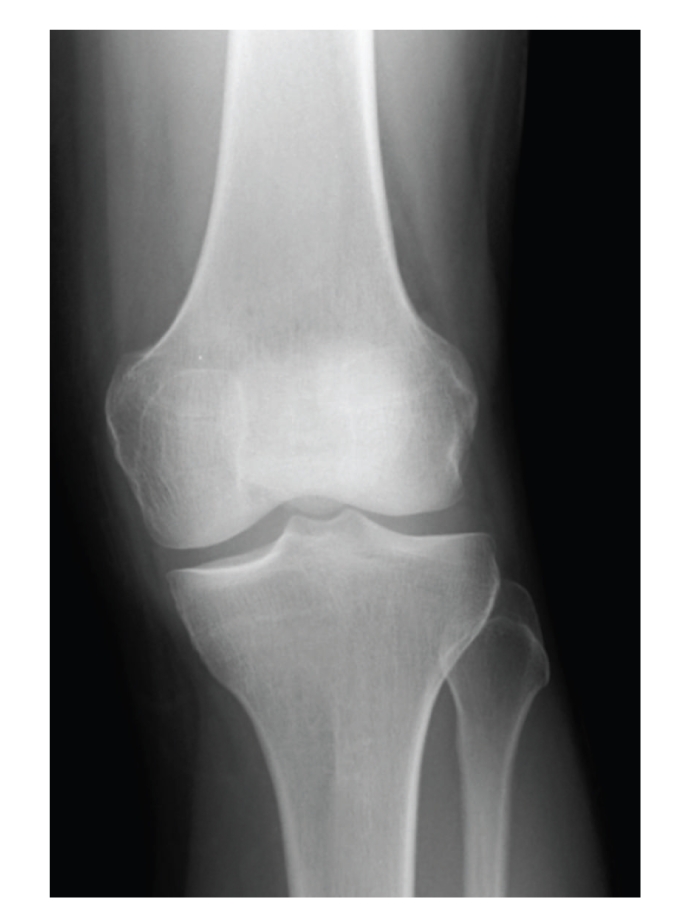
Normal AP radiograph of left knee.

**Figure 2:**
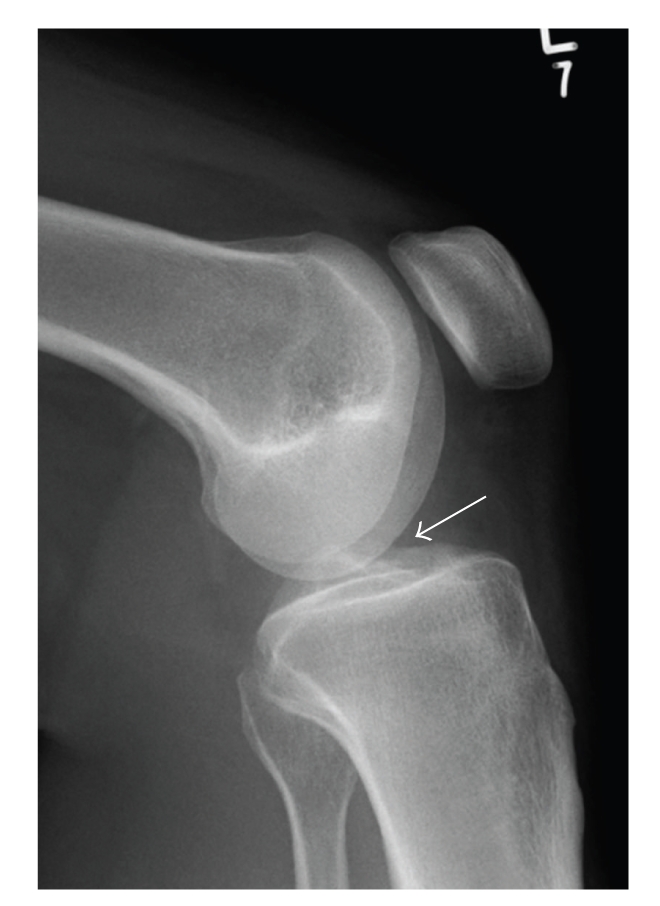
Lateral knee radiograph showing a soft tissue density lesion in the location of Hoffa's fat pad.

**Figure 3:**
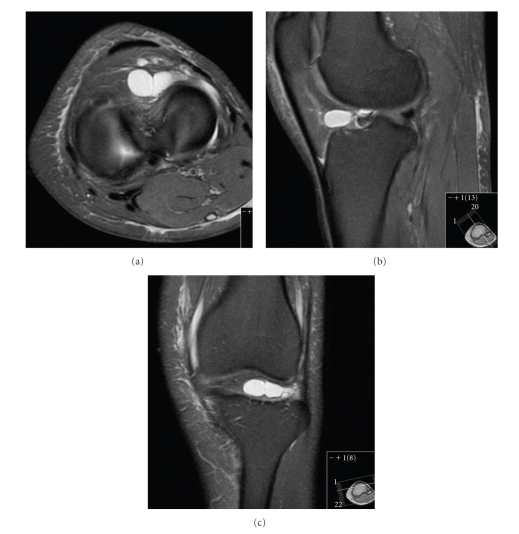
Axial/sagittal/coronal FD FS MRI showing a large multilocular cystic swelling arising from the ACL tibial footprint extending to the infrapatellar fat pad.

**Figure 4:**
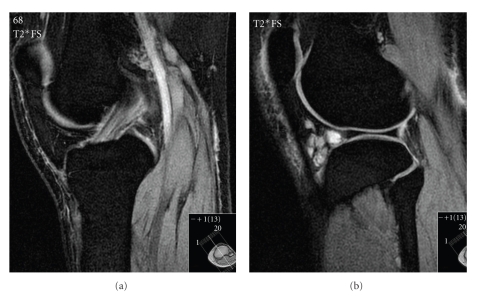
Para-sagittal and sagittal T2 FS MRI scans confirming a normal ACL signal and extensive extension of cystic lesion into Hoffa's fat pad.

**Figure 5:**
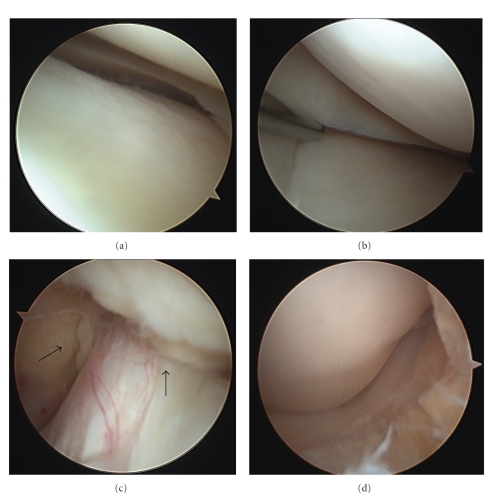
Intraoperative images displaying normal lateral (a) and medial (b) menisci, intact ACL (c) surrounded by debris, and the view of the anterior/lateral knee joint following extensive shaving into the fat pad (d).

Initial anteroposterior (AP) and lateral radiographs were reported as normal, but retrospectively a cystic swelling in the infrapatellar region could be suspected on the lateral view (Figures 1 and 2). Subsequent T2/STIR sequence magnetic resonance imaging (MRI) scans revealed a large high signal intensity lesion arising from the tibial footprint of the anterior cruciate ligament (ACL), though the ligament itself returned a normal signal. A significant extension of the lesion into the lateral infrapatellar fat pad was also seen. Since the menisci demonstrated no pathology, a radiological diagnosis of an intra-articular ACL ganglion cyst was made (Figures 3, 4, and 5).

The patient was reviewed 2 weeks after the MRI, at which point it was noted that her knee stiffness had progressed further such that only 20 degrees of flexion was elicitable. 

Under general anaesthesia the knee was examined, and an audible “pop” was noted on forced knee flexion. The range of movement was normalized, and no knee instability confirmed. A standard two-portal anterolateral and anteromedial arthroscopy was then conducted. Findings were of normal articular surfaces, normal ACL, and no meniscal pathology (Figure 5). No intact cyst was found, which is likely to have been burst by forcing the knee to 90° flexion to allow safe arthroscopy entry. Nevertheless debris from the ruptured wall and its contents were seen to extend into the lateral fat pad. Extensive suction assisted power shaving was conducted to remove this cystic material along with the surrounding fat pad (Figure 5). Postoperatively the patient underwent appropriate rehabilitation making an uneventful recovery with resolution of her pain and stiffness. Six months after surgery, the patient was symptom-free with no positive clinical signs and therefore MRI scan was not performed. 

## 3. Discussion

The first case of an intra-articular knee cyst was reported by Caan in 1929 following a cadaveric examination of an elderly male [8]. By the late 1980s, still only a handful of sporadic cases were described in the literature [9–13]. The last thirty years have seen a huge expansion in the number of these described cysts owing to the widespread use of both MRI and arthroscopy. The prevalence of knee intraarticular cystic masses is 1.3% on MRI and 0.6% following arthroscopy, with ganglia arising from the ACL predominating [4–7, 14]. The majority of these cysts are diagnosed incidentally and are thus asymptomatic. In MRI scans, these lesions appear as cystic masses with fluid signal intensity [15]. Ultrasound appearances are typically anechoic, and lesions may be either unilocular or multilocular [16]. Krudwig et al. have reported pain followed by stiffness as the most common presenting symptoms in 9 out of their 85 cases [4]. They have also noted that ACL cysts tend to produce a limitation in knee extension (locking), which is the converse of our case presentation (reverse locking). We postulate that the large extension into Hoffa's fat pad had produced a noncompliant anterior soft tissue wedge which could block knee flexion. The fact that the cyst ruptured on forced knee flexion would complement this hypothesis.

The pathogenesis of ganglion cyst formation remains controversial with theories including herniation of synovium into surrounding tissues, mucinous degeneration of connective tissue following trauma, or synovial displacement during embryogenesis [2]. The histological observation that these fluid filled structures have no epithelial lining confirms that they are not true cysts and therefore dispel the theories of synovial herniation favouring a degenerative cause [6].

The aetiology of the condition remains unknown. Despite many reports of ganglia developing in the absence of trauma, it is postulated that repetitive microtrauma from joint and soft tissue motion causes liberation of mucin/hyaluronic acid from ligament fibres and thus may act as the trigger [5].

Arthroscopic excision of ganglionic cysts has been shown to be the treatment of choice, with 95% of patients reporting good or excellent results [17]. Arthroscopic excision requires hospitalisation and general anaesthesia but has low recurrence rates [18, 19]. Percutaneous aspiration using computerised tomography (CT) scan and ultrasound guidance have advantages of being out-patient procedures with quicker recovery time [18]. PCL ganglion cysts have been successfully treated with CT and ultrasound-guided aspiration [18, 20] although recurrence rates are unknown. It has also been suggested that some cysts may reduce or disappear spontaneously with conservative management [21, 22].

## 4. Conclusion

Whilst rare, intra-articular ganglion cysts should be a differential diagnosis in cases of unexplained progressive knee pain or mechanical locking, particularly in the absence of overt trauma. This case highlights the importance of considering this diagnosis particularly in patients presenting with “reverse locking.” MRI is the investigation of choice for the identification of ganglia in the knee joint. The size, shape, and location can be accurately identified, thus aiding preoperative planning for arthroscopic resection, the treatment of choice.
